# The impact of digital inclusive finance on SMEs’ technological innovation activities—Empirical analysis based on the data of new third board enterprises

**DOI:** 10.1371/journal.pone.0293500

**Published:** 2023-11-02

**Authors:** Fuzhen Gu, Junguang Gao, Xiaoya Zhu, Jiamin Ye

**Affiliations:** 1 Economics and Management School, Panzhihua University, Panzhihua, Sichuan, China; 2 School of Business, Beijing Technology and Business University, Liangxiang, Beijing; Chongqing University, CHINA

## Abstract

The landscape of financial technology is undergoing a continuous evolution, driven by the relentless advancement of information technology. In this transformative milieu, digital finance has emerged as a novel financial paradigm, offering a blueprint for fostering inclusive finance. With a particular emphasis on its implications for Small and Medium-sized Enterprises (SMEs), this article harnesses a comprehensive dataset spanning the years 2011 to 2021, encompassing digital inclusive finance and SMEs listed on the SME board. Employing fixed effects models, this study performs a regression analysis to verify the driving effect of digital inclusive finance on SMEs’ innovation activities. The findings unequivocally affirm the potency of inclusive finance in ameliorating the longstanding financing constraints that have historically constrained the growth trajectory of SMEs. Furthermore, the study elucidated the nuanced nature of the promotional impact of digital inclusive finance on SMEs, contingent upon their distinct property rights and technological attributes. Specifically, the empirical findings unveil a discernible pattern wherein digital inclusive finance exerts a conspicuously stronger promotional effect on non-state-owned enterprises and high-tech SMEs’ endeavors in technological innovation. The conclusions derived from this research furnish a salient point of reference for governmental authorities engaged in the formulation and advancement of digital inclusive finance strategies, thereby imparting strategic guidance for the cultivation of innovation and holistic development within the SME sector.

## 1 Introduction

In the contemporary epoch characterized by the global new generation science and technology revolution, China finds itself immersed in a milieu of intense international competition within the realms of science and technology. This panorama unfolds against the backdrop of a pivotal juncture in its economic evolution [1]. Consequently, the imperatives of elevating China’s technological innovation prowess assume paramount significance and an escalating sense of urgency, converging with its overarching national ambition of ascending to a position of robust global prominence. This resolve is underscored by the “Outlines on National Strategy for Innovation-Driven Development,” a seminal document unveiled in 2016, which accentuates the pivotal role of enterprises as architects of innovation. Central to its implementation is the mandate to cultivate innovation subjects, an integral cornerstone of innovation-driven development strategy [2]. Amidst the existing landscape of the market economy system, SMEs emerged as quintessential and auspicious innovation entities. They possess the potential to evolve into a preeminent driving force, propelling the trajectory of technological innovation, effectuating transformative technological shifts, and assuming an increasingly pivotal role in the advancement of China’s national economic landscape [3]. This assertion is mirrored in the “Implementation Opinions on Promoting the Healthy Development of SMEs,” a proclamation unveiled in December 2019. This proclamation underscores the imperative of fortifying SMEs’ innovation capacity and expertise, heralded as a fundamental underpinning for propelling the trajectory of high-quality economic and social development [4]. However, for the sustenance of enduring innovative undertakings, SMEs are confronted with the imperative of accessing commensurate financial resources [5]. From a pragmatic standpoint, the pursuit of innovation is frequently intertwined with heightened risks, largely emanating from the inherent challenges of accurately anticipating innovation outcomes and the intricate discord between immediate returns and associated risks [6, 7].

Beyond the aforementioned challenges, the process of innovation transformation necessitates a substantial infusion of investment. This intricate journey entails substantial monetary outlays and a continuous inflow of resources, encompassing requisites such as equipment, human capital, and distribution networks [5, 8]. Additionally, the organizational framework and managerial ethos of an enterprise must harmonize seamlessly with the contours of the novel technology. Compounding the intricacy is the phenomenon of positive spillover inherent in innovation outcomes. This facet implies that the knowledge and technology germinated through the enterprise’s innovation pursuits are susceptible to emulation by peers and contemporaries, especially rival entities, thus endowing them with access to the economic fruits of innovation [9]. Consequently, this dynamic imparts a quirk of paradox, whereby financial institutions often find themselves disincentivized to extend financial provisions tailored to corporate innovation, thereby engendering a formidable impediment to the financing of innovation initiatives, particularly for SMEs [10].

SMEs, positioned as the long-tail constituents within the intricate fabric of the market economy system, find themselves particularly susceptible when embarking upon innovative financing undertakings. Their vulnerability stems from encountering formidable financing constraints, a consequence of their marginalization within the traditional bastions of financial institutions. Moreover, their forays into technological innovation often encounter limited efficacy within the conventional financial markets, further exacerbating their funding challenges [11]. In a transformative paradigm, however, the advent of digital finance has proven to be a resolute departure from the confines of conventional finance. By harnessing the potential of cloud computing technology, big data applications, and mobile interconnectivity, digital finance has transcended the limitations of its traditional counterpart [12]. This transformative wave has not only elevated the outreach of financial services but has also engendered a comprehensive and streamlined modern financial service ecosystem, characterized by pervasive accessibility, and heightened efficiency. In a seminal turn of events, the issuance of the “G20 Digital Inclusive Financial Principles” in 2016 heralded the ascendancy of digital finance to a universally acknowledged paradigm of financial inclusion. This watershed development instilled a catalyst for amplifying the innovation input of SMEs, infusing renewed vigor into their innovation pursuits [13, 14].

Considering this overarching backdrop, this article conducts an empirical expedition, forging a tangible link between the realms of digital inclusive finance, SME innovation endeavors, and the intricate fabric of financing constraints. The study’s primary thrust revolves around unraveling the intricate web of incentives nurtured by digital inclusive finance, particularly its role in catalyzing SMEs’ investments in technological innovation. A principal focus rests upon unveiling the operative mechanisms through which the burgeoning landscape of digital inclusive finance orchestrates a twofold transformation: the alleviation of the pervasive quandaries associated with financing constraints and the concomitant cultivation of a conducive milieu for augmenting SMEs’ investments in innovation, meticulously scrutinized at the microcosmic level of enterprises.

Given the intrinsic heterogeneity prevalent within the landscape of SMEs, which invariably begets a spectrum of financing constraints, this study undertakes a discerning exploration into the manifold ramifications of the operational mechanism. Delving into the intricacies of innovation across diverse SME typologies, this study adroitly probes this mechanism’s influence, casting a penetrating gaze through the prisms of property rights and technological attributes. The fundamental goal inherent in this investigation is to distill a reservoir of perspicacious insights and cogent decision-making benchmarks, thereby orchestrating a symphony of endeavors aimed at propelling the growth trajectory of digital inclusive finance. In tandem, this paper endeavors to fortify the bedrock of SMEs’ innovation capabilities, engendering a symbiotic partnership between digital inclusivity and enhanced innovative prowess.

## 2 Theory and hypotheses

### 2.1 The mechanism underpinning the impact of digital inclusive finance on SME innovation

The foundation of competence generation lies in the allocation and utilization of resources, as expounded by the resource-based theory [15, 16]. The spectrum of innovation endeavors mandates a continuous infusion of investment into R&D, spanning from the conceptualization of groundbreaking products to their successful commercialization [17]. However, the intricate plight faced by SMEs is that traditional financial institutions have historically shunned them due to their dearth of substantial collateral. Consequently, the attainment of requisite financial backing for innovation initiatives becomes a Herculean challenge [11]. Compounded by the inherent long-term nature and heightened uncertainty intrinsic to innovation pursuits, SMEs find themselves further hampered in securing innovation financing from conventional financial establishments [7, 8, 18]. This confluence of factors culminates in a discernible chasm in innovation investment [6]. Notable, statistical evidence underscores this predicament, revealing that SMEs are afforded access to a mere fraction (ranging from 1/4 to 1/3) of the overall financial resources, a stark disproportion compared to their significant economic contributions [19]. Evidently, the insufficiency of financial backing directed towards SME technological innovation reverberates as a pronounced hurdle, giving rise to acute scarcity in innovation investment.

In an era marked by the seamless integration of digital technology and financial services, barriers that have historically impeded the progress of financial services are gradually yielding ground [20]. Dispersed geographic locations and outdated infrastructural constraints, once formidable hurdles, are steadily being surmounted. A palpable consequence of this evolution is the relentless expansion of the financial services landscape [12, 21]. This expansion serves to alleviate the prevalent information asymmetry between the facets of fund supply and demand within the financial services domain [22]. Notably, this surge in coverage contributes to the amelioration of transaction costs, augments the efficacy of risk identification mechanisms within financial operations [12], and bolsters the credit ecosystem tailored for the benefit of SMEs [23, 24]. Evidently, this progressive evolution aligns more closely with the development requisites of SMEs [25]. This is underscored through the following salient dimensions:

The first impact mechanism of digital inclusive finance on SME innovation resides in its capability to diminish the barriers to entry for financial services, diversify avenues for financing, and augment reservoirs of funds for innovation investment [12, 20, 21, 24–26]. Digital inclusive finance adeptly amalgamates conventional financial instruments with Internet technology, surmounting the limitations inherent to traditional financial services, which are frequently curtailed by infrastructure constraints and geographical distance [20]. As a result, it effectively mitigates the threshold for accessing financial services, rectifies the incongruity between credit resources and demands, and augments the reach of financial services [24]. Moreover, digital inclusive finance facilitates the convergence of credit and capital markets, nurturing inventive credit and equity-backed products, and affording the dynamic tailoring of financing dimensions, modalities, and temporal alignment [23]. This orchestration not only enhances the alignment between funding supply and requisites but also underscores judicious resource allocation and amplifies the comprehensive utilization of financial services. This orchestrated synergy demonstrably mitigates the financing bottlenecks that typically encumber SME innovation. In consequence, digital inclusive finance engenders a panorama of enhanced prospects and choices for SME innovation funding investment, thereby catalyzing the spectrum of possibilities for SMEs in their pursuit of innovative endeavors.

The second conduit by which digital inclusive finance propels SME innovation rests in its capacity to mitigate the quandary of information asymmetry between financial institutions and SMEs, thereby engendering a decline in the outlay associated with innovation financing [26]. With the objective of enhancing transparency in business intelligence and curtailing transaction expenses, conventional financial entities have instituted digital modalities such as online banking and mobile banking [24]. Additionally, the digital milieu has precipitated the emergence of fully virtual banks and P2P online lending platforms, contributing to the seamless dissemination of intelligence to SMEs pursuing financial support [27]. This evolution significantly curtails the outlays related to both search and transaction operations. Digital inclusive finance capitalizes on internet technologies such as cloud computing, search engines, and social networks, endowed with unparalleled information aggregation and processing prowess [12]. This technological framework effectively supplants labor-intensive manual procedures, engendering reductions in manual processing and coordination outlays, alongside the costs affiliated with risk evaluation in lending activities [26]. Consequently, it attains a discernible decrease in the expense incurred for SMEs to access innovation-driven financing, thus augmenting the feasibility of SMEs to harness financial support for their innovative pursuits.

The third instrumental mechanism facilitated by advanced digital technology involves financial institutions harnessing expansive datasets from diverse platforms. This data-driven approach encompasses the gathering, organization, and analysis of behavioral data spanning various industries and enterprises. This methodology efficaciously mitigates the predicaments of inadequate credit and financial information, along with the elevated credit default risk that SMEs encounter when soliciting funds from banks [13, 28]. Leveraging digital technology, financial institutions adeptly amass and correlate behavioral data pertinent to SMEs, thus enabling multi-faceted credit assessments of target entities [13, 23]. This novel credit evaluation framework instills confidence among guarantors, thereby underpinning the provision of financial backing to SMEs [24, 29]. Furthermore, the introduction of approval loan technology, rooted in digital advancement, streamlines the qualification appraisal and loan sanctioning process for enterprises. This innovation effectively counteracts the historically disadvantageous financing policies for SMEs, concurrently bolstering the efficiency of innovative funding endeavors [23, 29]. By optimizing the assessment processes and mitigating inefficiencies, digital technology redefines the landscape of SME financing, enhancing access and expediting support.

Building upon the manifold benefits of digital inclusive finance, which encompass its extensive coverage, reduced transaction costs, and heightened capital efficiency, the debilitating financing constraints that have hampered SMEs find effective mitigation [30]. With its inherently inclusive nature, digital finance galvanizes SMEs with an expanded array of financing opportunities, thus affording them a sustained and unwavering means to address their ongoing funding requisites for technological innovation activities. Guided by these insights, this article posits the following two hypotheses:

**Hypothesis 1:** Digital inclusive finance exerts a positive driving effect in fostering technological innovation activities among SMEs.
**Hypothesis 2:** By alleviating the financing constraints faced by SMEs, digital inclusive finance propels and sustains their technological innovation endeavors.

### 2.2 Heterogeneous analysis of the impact of digital inclusive finance on innovation among different types of SMEs

Within the research sample comprising SMEs, individual firms exhibit inherent characteristics that set them apart, and diverse categories of SMEs may encounter varying institutional influences in their financing endeavors [31, 32]. Consequently, the influence of digital inclusive finance on investment in technology innovation might manifest distinctively across diverse SME classifications. To address this, this study segments the sample based on property rights and technological innovation within distinct SME types, catalyzed by the intervention of digital inclusive finance.

#### 2.2.1 Property rights of SMSs

Examining the property rights of SMEs unveils significant distinctions in the financing landscape between non-state-owned and state-owned entities [33]. The ownership framework of enterprises plays a pivotal role in dictating the distribution of financial resources within the market. Notably, financing constraints confronting SMEs diverge in accordance with their ownership composition [31, 32]. In particular, non-state-owned businesses, in contrast to their state-owned counterparts, contend with issues of information opacity and incomplete financial disclosure, contributing to information asymmetry quandaries between enterprises and financial institutions [22]. In the prevailing Chinese societal framework, non-state-owned SMEs grapple with formidable hurdles such as “financing difficulties and elevated borrowing costs,” stemming from conventional financial establishments’ tendency toward credit bias [29].

Against the backdrop of advancing digital inclusive finance, financial institutions harness emergent technologies such as cloud computing and big data analytics to undertake comprehensive assessments of enterprises’ credit standing, financial health, and operational status. This endeavor, in turn, mitigates information asymmetry and ameliorates financing limitations experienced by non-state-owned SMEs [32]. Moreover, SMEs of varying ownership structures exhibit disparate levels of reliance on digital inclusive finance [29]. State-owned enterprises assume augmented social responsibilities in the modern economic milieu and are intertwined with national macroeconomic strategies [34]. Consequently, state-owned entities typically exhibit larger scales and well-structured internal governance systems, translating into comparatively comprehensive internal financial information.

Additionally, discernible disparities in strategic orientation and organizational management models between non-state-owned and state-owned enterprises come to the fore [31, 32]. This divergence positions state-owned businesses to potentially secure financial backing from state-owned banks and governments at relatively diminished borrowing costs, even in the face of liquidity crises or deficits [35, 36]. As China’s financial market milieu gradually evolves and multi-tiered capital markets gain traction, enterprises’ access to financing channels will diversify, exerting a more pronounced influence on non-state-owned entities’ operational and financing choices [37].

Moreover, scholarship implies that state-owned enterprises exhibit relative inflexibility in their operational structure and manifest a lack of adaptability [38], potentially impeding a smooth transition from reliance on government relations as financing conduits to embracing the channels of digital inclusive finance. Conversely, the financing attributes synonymous with digital inclusive finance, characterize by simplicity, speed, and suitability for modest sums [29], align more congruently with s the financing requisites of non-state-owned SMEs, which frequently exhibit comparatively modest funding needs [26]. Grounded in this discourse, the study posits the formulation of the third hypothesis:

**Hypothesis 3:** The impact of digital inclusive finance on innovation investment varies across SMEs with distinct ownership structures, with its influence more pronounced in fostering innovation investment among non-state-owned SMEs.

#### 2.2.2 Technological characteristics of SMEs

Delving into technical attributes, a conspicuous dichotomy emerges in the R&D intensity and funding constraints observed between high-tech and non-high-tech SMEs [39]. High-tech SMEs, occupying the terrain of knowledge- and technology-intensive sectors, navigate swiftly evolving industry landscapes and product substitutions. Their innovation initiatives constitute linchpins of revenue generation, propelling augmented investment in R&D to bolster technological advancement [40]. Notably, these innovation undertakings necessitate sustained capital outlays, underscoring the challenge faced by such SMEs in wholly relying on internal financing to meet funding imperatives [5]. Consequently, high-tech SMEs turn to external financing avenues, yet confront accentuated constraints due to the heightened risk and protracted lifecycles characterizing innovation endeavors [41]. In this context, digital inclusive finance materializes as a panacea to funding conundrums besieging high-tech SMEs. Digital inclusive finance surmounts the limitations tethered to conventional financial institutions’ lending practices, employing digital information surveillance to encompass multidimensional credit funds flows across temporal horizons [20, 30]. Furthermore, by adroitly scrutinizing high-tech SMEs’ enterprise value and growth trajectories, digital inclusive finance competently foresees and assesses potentialities, curtailing information asymmetry and collateral insufficiencies inherent in the financing trajectory. Ergo, the impact of financing constraints mitigation gains pronounced traction, precipitating a heightened proclivity for innovation within high-tech SMEs [23, 42]. Grounded in this scrutiny, this paper postulates the formulation of the fourth hypothesis:

**Hypothesis 4:** Digital inclusive finance engenders disparate impetuses on the innovation investment tendencies of SMEs hinging on their technological attributes, wielding a more conspicuous sway in augmenting innovation investment among high-tech SMEs.

## 3 Methodology

### 3.1 Sample

This study centers on the examination of China’s listed SMEs, with a temporal scope encompassing the years spanning from 2011 to 202l. The dataset employed to gauge digital inclusive finance derives from the CSMAR database, wherein meticulous data refinement procedures were instituted. This entailed the culling of delisted entities, companies evincing anomalous or incomplete data, and financial enterprises. To enhance data fidelity, a 2.5% truncation process was employed, targeting continuous variables within the sample dataset to attenuate the influence of outliers. Consequent to these measures, an unbalanced panel data set of 6543 SMEs emanating from 862 companies materialized, serving as the bedrock for the ensuing investigation.

### 3.2 Variables

#### Dependent variable

In gauging enterprises’ innovation capability and prowess, extant scholarly literature outlines three principal methodological avenues. The first entails the selection of an innovation input index as a gauge of enterprises’ innovation capacity. This index comprehensively mirrors SMEs’ subjective inclination toward participating in innovative undertakings and serves as a proxy indicator for the technological innovation extent of these entities [43, 44]. Pertinent variables measuring innovation investment encompass firms’ R&D expenditures [45], with indicators like aggregate R&D outlays and the number of R&D personnel chiefly reflecting this expenditure [46].

The second method revolves around employing innovation output indicators to quantify firms’ innovation capacity. Each such output indicator, to a certain degree, symbolizes a firm’s technological innovation competence [47]. Notable indicators for gauging innovation output encompass the valuation of novel products and the number of patents secured [48]. Scholars commonly opt for the number of patents as a gauge of a firm’s technological innovation prowess, asserting its capacity to truly reflect the core technological innovation level [49, 50]. However, differing interpretations emerge regarding the assessment of patent indicators. Some advocate for utilizing the count of patent applications for measurement [51], while others opt for the count of granted patents [52]. Further perspectives posit that evaluating firms’ innovation capability from a single dimension is less objective and comprehensive and that a dual-output dimension affords greater precision [53].

Moreover, the multifaceted factors impacting firms’ innovation levels necessitate consideration [54]. These factors span not solely internal innovation inputs but encompass diverse external environmental elements—government policies, and market recognition—beyond firms’ immediate control [55]. In this study, centered on appraising the influence of financial elements on SMEs’ innovation levels, *innovation input* is employed to gauge the latter [56]. Specifically, *the total R&D expenditure of enterprises* serves as a proxy variable to encapsulate firms’ innovation input levels. To ensure scale uniformity, the R&D expenditure indicator is dimensionless and presented as a ratio of total R&D expenditure to total operating revenue for the given year [57].

#### Independent variables

To investigate the influence of digital inclusive finance on SMEs’ innovation capacity, we incorporate the Digital Inclusive Finance Index (DIFI), established by the Digital Finance Research Center of Peking University, as the principal explanatory variable [58]. This index encompasses four constituent indices: the total DIFI, the Coverage Breadth Index (DUD), the Usage Depth Index (DSS), and the Digital Support Services Index (DCB). Collectively, these indices offer a comprehensive depiction of the impact of digital technology on financial innovation, illuminating the degree of digital inclusive finance development in China [58]. Renowned for their robust representativeness, dependability, and credibility, these indices have featured prominently in research endeavors pertaining to digital finance. In this investigation, we employ the data for each prefecture-level city during the period spanning 2011–2021 as proxy variables for the indicators of the digital inclusive finance index. These proxies effectively measure the level of digital inclusive finance development. It is noteworthy that the data for this suite of financial index indicators align temporally with the data pertaining to the dependent variable.

#### Mediating variable

Due to the intrinsic attributes of high investment and elevated risk associated with innovation, conventional financial frameworks encounter challenges in adequately catering to the capital requisites of small and medium-sized enterprises (SMEs) in pursuit of innovative endeavors. The pronounced constraints on financing exert a profound inhibitory influence on the capacity of SMEs to engender technological innovation. In light of contemplating the potential of digital inclusive finance to ameliorate the quandary of "financing difficulty" confronted by SMEs, the present study opts to position financing constraints as an intermediary construct. This strategic choice propels a deeper exploration into the potential of digital inclusive finance to facilitate technological innovation within SMEs, mediated through the mitigation of financing constraints.

Within the purview of assessing financing constraints, diverse indices are extant, among which the KZ Index [59], WW Index [60], and SA Index [61] rank prominently. In juxtaposition to the KZ and WW Indices, the SA Index distinguishes itself by furnishing several methodological advantages in quantitatively expounding the rigidity of financing constraints. The construction of the SA Index entails variables characterized by limited temporal volatility and robust exogeneity. Consequently, the deployment of such variables is poised to mitigate endogeneity biases that might obfuscate empirical findings. Secondly, grounded in considerations of data availability, the constituent sub-indicators of the SA Index manifest greater tractability with respect to data collection and computational expediency. Thirdly and conclusively, the SA Index evinces a heightened resilience within its estimations.

Accordingly, the present research underscores the salience of the SA Index as a surrogate metric for gauging the magnitude of financing constraints. Aligned with the methodological praxis advocated by Hadlock & Pierce (2009), the SA Index is computed as SA = -0.737 × Size + 0.043 × Size^2–0.04 × Age. In this formulation, ’Size’ denotes the organizational scale, while ’Age’ encapsulates the temporal trajectory of the enterprise (as expounded upon in the definitional exegesis furnished in Table 1).

**Table 1:** Main variables definition table.

Variable Type	Variable Symbols	Variable Name	Variable Description
Dependent variable	INNOV	*Enterprise Innovation Investment*	(the total R&D expenditure/ operating revenue)*100%
Mediating variable	SA	*Financing constraint index*	Based on Hadlock&Pierce(2009)’s constructing method
Control variables	SIZE	*Enterprise size*	Ln (the total assets at the end of the period)
	ROA	*Asset profitability*	Net profit/Total assets
	GROW	*Enterprise growth*	Ratio of current year’s revenue change to previous year’s revenue
	FAS	*Share of fixed assets*	End-of-period fixed assets/End-of-period total assets
	Age	*Enterprise age*	Natural logarithm of the difference between observation year and establishment year
	Ind	*Proportion of independent directors*	Number of independent directors/Total number of directors
	Dual	*CEO Duality*	1 for Chairman and CEO duality, 0 for separation of roles
	LEV	*enterprise asset-liability ratio*	the total liabilities of the enterprise at the end of the year / the total assets at the end of the year
Independent variables	DIFI	*The digital inclusive finance index*	Peking University Digital Inclusion Index
	DSS	*usage depth index*	
	DUD	*coverage breadth index*	
	DCB	*Digital Support Services Index*	

#### Control variables

Recognizing the potential influence of internal factors within enterprises on their innovation capacities and building upon pertinent literature on financial development and technological innovation in enterprises [2, 29], this study aims to mitigate potential endogeneity biases originating from omitted variables. Consequently, the model integrates various control variables into analysis, encompassing enterprise size (SIZE), return on assets (ROA), enterprise growth (GROW), fixed asset share (FAS), enterprise age (Age), the proportion of independent directors (Ind), dual identity of chairman and CEO (Dual), and asset-liability ratio (Lev). Additionally, the analysis incorporates annual dummy variables and industry-specific dummy variables (refer to Table 1 for precise definitions of all variables).

### 3.3 Model construction

The principle aim of this study is to investigate the potential influence of digital inclusive finance on innovation investment in SMEs. The dependent variable is represented by innovation investment (INNOV), while the independent variables consist of the digital inclusive finance index (DIFI) and its constituent sub-indices (DCB, DUD, DSS). Furthermore, the model includes several control variables, encompassing enterprise size (SIZE), return on assets (ROA), enterprise growth (GROW), fixed asset share (FAS), enterprise age (Age), independent director ratio (Ind), two-job combination (Dual), and asset-liability ratio (Lev). The model is structured as follows:

$$INNON_{i,\;t}=\alpha +\beta_{1}{\mathrm{DIFI}}_{i,\;t}+\beta_{2}DUD_{{}_{i,\;t}}+\beta_{3}DSS_{i,\;t}+\beta_{4}DCB_{i,\;t}+\sum \varphi CV_{i,\;t}+\sum Y\mathrm{ear}+\sum \mathrm{Industry}+\varepsilon$$ (1)

In the provided equations, the variables *i* and *t* correspond to the enterprise and year, respectively. The constant term is denoted as *α*, while *ε* represents the error term. The coefficients are represented by *β*_1_,*β*_2_,……*β*_7_. The dependent variable, *INNOV*, signifies the level of innovation investment undertaken by the firm. The explanatory variables consist of the total digital finance index (DIFI) along with its sub-indices (DCB, DUD, DSS). Moreover, CV represents the set of control variables mentioned earlier. To mitigate the impact of the industry in which the enterprise is located on innovation, this study uses a fixed effects model, controlling for time effects (Year) and industry effects (Industry).

In order to gain a deeper understanding of the mechanism through which digital inclusive finance influences innovation in small and medium-sized enterprises (SMEs), the present study introduces financing constraints (denoted as SA) as an intermediary variable. The absolute value of SA is employed as a proxy indicator for financing constraints. This approach is undertaken to empirically investigate whether digital inclusive finance facilitates the promotion of technological innovation activities within SMEs by alleviating their financing constraints. The research employs a stepwise approach to examine and analyze the influencing mechanism of how digital inclusive finance fosters innovation within SMEs. The regression model is specified as follows:

$$INNON_{i,\;t}=\alpha_{0}+\alpha_{1}{\mathrm{DIFI}}_{i,\;t}+\sum \varphi CV_{i,\;t}+\sum Y\mathrm{ear}+\sum \mathrm{Industry}+\varepsilon$$ (2)

$$SA_{i,\;t}=\beta_{0}+\beta_{1}{\mathrm{DIFI}}_{i,\;t}+\sum \varphi CV_{i,\;t}+\sum Y\mathrm{ear}+\sum \mathrm{Industry}+\varepsilon$$ (3)

$$INNON_{i,\;t}=\gamma_{0}+\gamma_{1}{\mathrm{DIFI}}_{i,\;t}+\gamma_{2}SA_{i,\;t}+\sum \varphi CV_{i,\;t}+\sum Y\mathrm{ear}+\sum \mathrm{Industry}+\varepsilon$$ (4)

In Eqs (2), (3), and (4), where "i" and "t" represent enterprises and years respectively, "*α*_0_,*β*_0_,*γ*_0_" represents the intercept term, "ε" denotes the error term, and "*α*_1_,*β*_1_,*γ*_1_,*γ*_2_" signifies the regression coefficients. The variables and indicators are defined as described in the preceding text. According to the principles of the mediation effects model, the total effect “*α*_1_”, direct effect “*γ*_1_”, and indirect effect “*β*_1_*γ*_2_” should satisfy the following equation: *α*_1_ = *γ*_1_+*βγ*_2_. In consideration of the theoretical analysis and research assumptions outlined earlier, if the coefficient of "*α*_1_" is positive, it signifies that the development of digital inclusive finance, on the whole, fosters the advancement of technological innovation in SMEs. Concurrently, if the coefficient of "*β*_1_" is negative, it indicates that the development of digital finance mitigates the financing constraints faced by SMEs. Furthermore, if the coefficient of "*γ*_2_" is negative, it implies that the reduction of financing constraints can enhance the level of technological innovation among SMEs [62].

## 4 Data analysis and results

### 4.1 Descriptive statistics of variables

This section presents descriptive statistics for the variables under investigation, with summarized findings presented in Table 2. In terms of innovation input (Innov), the mean value is 4.6271, accompanied by a median of 3.7400 and a standard deviation of 3.5887. The substantial range between the minimum (0.1500) and maximum values (16.8700) indicates significant diversity in the level of innovation input across the sample enterprises. Regarding the Digital Inclusive Financial Index (DIFI), the mean value stands at 2.2909, with a median of 2.3896 and a standard deviation of 0.7160. notably, the minimum value is 0.7762, while the maximum value is 3.4334, underscoring the considerable disparities in digital inclusive financial development across various cities.

**Table 2:** Statistical description of the variables.

VarName	Obs	Min	Median	Mean	Max	SD
INNOV	6543	0.1500	3.7400	4.6271	16.8700	3.5887
DIFI	6543	0.7762	2.3896	2.2909	3.4334	0.7160
DUD	6543	0.8087	2.3611	2.2859	3.5724	0.7176
DSS	6543	0.7653	2.4036	2.2530	3.2768	0.7146
DCB	6543	0.3972	2.5840	2.3740	3.2950	0.8216
size	6543	20.3495	21.8896	21.9578	24.1158	0.9156
ROA	6543	-0.1494	0.0383	0.0381	0.1523	0.0553
GROW	6543	-0.0041	0.0013	0.0024	0.0173	0.0043
FAS	6543	0.0001	0.0019	0.0021	0.0050	0.0012
Age	6543	6.0000	16.0000	16.0827	28.0000	5.4234
Ind	6543	0.3333	0.3333	0.3738	0.5000	0.0489
Dual	6543	0.0000	0.0000	0.3448	1.0000	0.4753
Lev	6543	0.0825	0.3734	0.3818	0.7533	0.1783

### 4.2 Baseline regression results

This study employs regression analysis to explore the relationship between the development of digital inclusive finance and the innovation level of SMEs. The explanatory variables consist of the total digital inclusive finance index and individual dimension indices. The regression outcomes, as presented in Table 3, reveal noteworthy patterns. Specifically, *the total digital inclusive finance index* (DIFI), *the coefficient of coverage breadth index* (DUD), *the usage depth index* (DSS), and *the digital service support index* (DCB) all exhibit positive coefficients. Furthermore, the associated p-values for these variables are less than 0.01 or 0.05, underscoring their statistically significant and positive effects. These empirical results, detailed in Table 3, offer robust support for Hypothesis 1. The findings underscore that the advancement of digital inclusive finance significantly and positively influences the innovation input level within SMEs. This implies that the promotion of digital inclusive finance can play a pivotal role in augmenting innovation within SMEs.

**Table 3:** Baseline regression results.

VARIABLES	(1)	(2)	(3)	(4)
	INNOV	INNOV	INNOV	INNOV
DIFI	1.0708***			
	(5.7302)			
DUD		0.7940***		
		(5.4371)		
DSS			0.8070***	
			(5.0924)	
DCB				0.5697**
				(2.4190)
size	0.1779***	0.1788***	0.1771***	0.1757***
	(3.3885)	(3.4050)	(3.3698)	(3.3350)
ROA	-6.1860***	-6.1650***	-6.1749***	-6.0657***
	(-7.3242)	(-7.2996)	(-7.3122)	(-7.1857)
GROW	80.7939***	80.2384***	81.2711***	78.9844***
	(8.1280)	(8.0744)	(8.1515)	(7.9319)
FAS	-51.3210	-51.8848	-61.1990*	-76.3453**
	(-1.4465)	(-1.4535)	(-1.7442)	(-2.1704)
Age	-0.0105	-0.0103	-0.0112	-0.0111
	(-1.4094)	(-1.3830)	(-1.4972)	(-1.4737)
Ind	0.4955	0.4669	0.6277	0.6865
	(0.6599)	(0.6203)	(0.8380)	(0.9150)
Dual	-0.0741	-0.0723	-0.0641	-0.0367
	(-0.9789)	(-0.9543)	(-0.8475)	(-0.4849)
Lev	-4.9365***	-4.9537***	-4.8694***	-4.8640***
	(-17.5960)	(-17.6324)	(-17.3763)	(-17.3241)
Constant	-1.2304	-1.2098	-1.1105	-1.1701
	(-1.0852)	(-1.0651)	(-0.9779)	(-1.0266)
Observations	6,543	6,543	6,543	6,543
R-squared	0.435	0.435	0.434	0.432
Industry FE	YES	YES	YES	YES
Year FE	YES	YES	YES	YES

Robust t-statistics in parentheses

*** p<0.01

** p<0.05

* p<0.1

Table 3 displays the outcomes of the regression analysis for the control variables, which generally align with the anticipated patterns. The coefficient associated with enterprise size is notably positive, signifying a favorable correlation between enterprise size and innovation capabilities. Larger enterprises tend to benefit from enhanced access to social resources and a wider array of financing avenues. This facilitates their capacity to prioritize long-term growth and allocate substantial resources to innovation investment, thereby bolstering their innovation capabilities. Similarly, the coefficient linked to enterprise growth demonstrates a significant positive correlation. This suggests that enterprises experiencing growth tend to exhibit heightened levels of innovation capabilities. This phenomenon can be attributed to the dynamic nature of growing enterprises, which necessitates ongoing innovation efforts to sustain their growth trajectory.

Conversely, the coefficient of return on assets (ROA) displays a significant negative relationship. This implies that enterprises with more robust profitability might display reduced inclination and incentive for innovation. Additionally, these enterprises could be constrained by external financing limitations, prompting them to adopt a more cautious approach toward innovation investment. Instead, they might prioritize retaining profits for internal use rather than engaging in innovative endeavors. Furthermore, the coefficient linked to leverage (Lev) showcases a significant negative correlation. Enterprises carrying higher leverage encounter difficulties in meeting repayment obligations, which subsequently curtails their access to innovative financing opportunities. Consequently, these enterprises are prone to exhibit diminished levels of investment in innovation. Cumulatively, the regression analysis pertaining to the control variables contributes valuable insights into the determinants influencing the innovation capabilities of SMEs.

### 4.3 Mediating effect of financing constraints regression results

In accordance with the testing procedure of the intermediary effect, the initial step involves scrutinizing the aggregate impact of inclusive digital finance on innovation within the realm of SMEs. The outcomes of this analysis, delineated in the first column of Table 4, reveal a notably positive and statistically significant regression coefficient at the 1% level of significance. Subsequently, an inquiry into the interconnectedness of inclusive digital finance and the financial constraints confronting SMEs ensues. The regression findings, presented in the second column of Table 4, manifest a statistically significant negative coefficient, reaching the 1% threshold of significance. This outcome substantiates a discernible inverse correlation between the extent of inclusive digital finance engenders a broader spectrum of financing alternatives for SMEs, thus mitigating, to some extent, the overarching predicaments related to financing constraints. Lastly, discerning the regression results within the third column of Table 4, it is observed that the coefficient pertaining to the intermediary variable denoted as “SA” is both negative and statistically significant at the 5% level. This observation underscores the role of financing constraints as a partial mediating mechanism, explicating the conduit through which the advancement of inclusive digital finance impels innovation within SMEs. Consequently, this serves to validate Hypothesis 2, as posited in the present study.

**Table 4:** Mediating effect of financing constraints regression results.

	(1)	(2)	(3)
VARIABLES	INNOV	SA	INNOV
DIFI	1.0708***	-0.0121***	1.0692***
	(5.7302)	(-3.1079)	(5.6334)
SA			-0.1344**
			(-2.2360)
size	0.1779***	0.0162***	0.1801***
	(3.3885)	(10.7892)	(3.4447)
ROA	-6.1860***	0.0241	-6.1827***
	(-7.3242)	(1.6049)	(-7.3212)
GROW	80.7939***	0.2891*	80.8328***
	(8.1280)	(1.6715)	(8.1278)
FAS	-51.3210	-3.4122***	-51.7797
	(-1.4465)	(-4.5089)	(-1.4602)
Age	-0.0105	0.0401***	-0.0051
	(-1.4094)	(277.2522)	(-0.2123)
Ind	0.4955	-0.0728***	0.4857
	(0.6599)	(-4.0060)	(0.6451)
Dual	-0.0741	0.0020	-0.0738
	(-0.9789)	(1.2840)	(-0.9744)
Lev	-4.9365***	0.0113**	-4.9350***
	(-17.5960)	(2.5033)	(-17.5852)
Constant	-1.2304	2.8032***	-0.8536
	(-1.0852)	(78.5336)	(-0.4145)
Observations	6,543	6,543	6,543
R-squared	0.435	0.941	0.435
Industry FE	YES	YES	YES
Year FE	YES	YES	YES

Robust t-statistics in parentheses

*** p<0.01

** p<0.05

* p<0.1

The discernible reduction in the coefficient associated with the nexus between digital inclusive finance and innovation, upon the inclusion of considerations pertaining to financing constraints, can be ascribed to the prevailing reliance of a majority of SMEs upon constricted internal financial resources. Consequently, these entities encounter they encounter impediments in bridging the financing chasm requisite for the sustenance of their R&D activities [63]. In contexts where the avenues for external funding exhibit fragility, SMEs exhibit a tendency to curtail their investments earmarked for novel product development, thereby exerting a dampening influence upon their innovation pursuit. The innovative framework propagated by the paradigm of digital inclusive finance bestows a suite of advantages that adeptly mitigate the challenges at hand. This framework efficaciously ameliorates the disparities in information between conventional financial institutions and SMEs, streamlines the labyrinthine pathways of approval processes, and augments the overall efficiency of the financing mechanisms. Additionally, the integration of cutting-edge big data technologies serves to redress the paucity of credit-related information pertinent to SMEs, relaxes the strictures associated with collateral prerequisites, and expedites their access to supplementary funding sources [23, 24, 29]. Consequently, the evolution of digital inclusive finance substantially alleviates the fiscal constrictions that beleaguer SMEs, thereby engendering a marked advancement in their innovation endeavors [64].

### 4.4 Heterogeneity analysis

In order to scrutinize the divergent effects of digital inclusive finance on investments in innovation across diverse enterprise types, this study employs a classification framework rooted in property rights and technological attributes for the purpose of categorizing SMEs. Subsequent to this classification, a collective regression analysis is undertaken, with the ensuing outcomes being meticulously delineated in Table 5.

**Table 5:** Results of heterogeneity testing based on property rights and technological characteristics.

VARIABLES	State-owned SMEs	Non-state-owned SMEs	Hi-tech SMEs	Non-hi-tech SMEs
	(1)	(2)	(3)	(4)
	GQINNOV	FGQINNOV	GXINNOV	FGXINNOV
DIFI	1.0917	1.3605***	1.0901***	0.8940**
	(1.5589)	(7.2247)	(4.9990)	(2.5486)
size	0.4522***	0.0985*	0.0490	0.5146***
	(3.3909)	(1.6843)	(0.7850)	(5.3488)
ROA	-3.6674	-6.9364***	-7.9012***	-2.6718*
	(-1.5384)	(-7.6470)	(-7.6267)	(-1.7992)
GROW	17.3538	85.6205***	72.6519***	105.9842***
	(0.8002)	(7.5287)	(5.6099)	(6.0242)
FAS	-402.0407***	16.9102	-96.7793**	-105.6760*
	(-4.6958)	(0.4325)	(-2.1616)	(-1.7185)
Age	-0.0604**	-0.0104	-0.0175**	0.0105
	(-2.3391)	(-1.2872)	(-1.9868)	(0.7241)
Ind	-2.7110	0.9891	2.9027***	-4.0616***
	(-1.0691)	(1.2371)	(3.0192)	(-3.1622)
Dual	0.6486**	0.0573	0.1772*	-0.6081***
	(2.1780)	(0.7297)	(1.8418)	(-4.5423)
Lev	-7.0230***	-4.8044***	-5.3004***	-4.0150***
	(-8.6381)	(-15.5807)	(-16.1147)	(-7.1773)
Constant	-3.8174	-0.0536	-0.2893	-7.3535***
	(-1.3640)	(-0.0421)	(-0.1997)	(-3.5039)
Observations	1,010	5,533	4,133	2410
R-squared	0.578	0.432	0.376	0.574
Industry FE	YES	YES	YES	YES
Year FE	YES	YES	YES	YES

Robust t-statistics in parentheses

*** p<0.01

** p<0.05

* p<0.1

#### 4.4.1 Analysis of heterogeneity in property rights

The research findings, outlined in Table 5, particularly focusing on columns (1) and (2), reveal the evident impacts of digital inclusive finance on innovation in different categories of SMEs. These categories are differentiated by ownership, specifically state-owned and non-state-owned entities. For non-state-owned SMEs, the regression analysis highlights a significantly strong influence of the comprehensive digital finance index. This influence is statistically significant even at a high threshold of 1% significance. This robustly suggests a clear link between digital inclusive finance and the promotion of innovative dynamics within these businesses. However, contrasting results emerge from the regression analysis pertaining to state-owned SMEs. The previously established conclusion doesn’t apply to this specific group. This divergence can be attributed to the varying degrees of financial challenges faced by SMEs with different ownership types. Non-state-owned SMEs, in particular, experience greater difficulties accessing traditional finance services. This leads them to seek funding through alternative means more intensely. These empirical findings validate the proposition presented in Hypothesis 3, which asserts that the influence of digital inclusive finance on innovation investment varies based on the unique characteristics of SMEs with different ownership structures. It’s noteworthy that this influence is especially pronounced in driving innovation investment within the realm of non-state-owned SMEs.

#### 4.4.2 Analysis of heterogeneity in technological features

Columns (3) and (4) within Table 5 undertake an examination of the heterogeneous effects of digital inclusive finance on innovation across two distinct cohorts of SMEs: those classified as high-tech and those categorized as non-high-tech. The outcomes of the regression analyses reveal a pervasive enhancement of innovation engendered by digital inclusive finance in SMEs at large. However, it is noteworthy that the affirmative impact of digital inclusive finance on innovation within the high-tech SMEs segment is conspicuously more pronounced. This finding intimates a nuanced stratification in the efficacy of digital inclusive finance as a driver of innovation, contingent upon the technological attributes inherent in SMEs. The discernibly heightened influence on innovation augmentation within high-tech SMEs stands as a salient observation. In so doing, these empirical observations lucidly buttress the assertions postulated in the fourth hypothesis.

### 4.5 Robustness test and endogeneity handling

#### 4.5.1 Robustness test

In order to fortify the integrity of the findings, this study employed two rigorous tests for robustness. Firstly, the dependent variable underwent substitution with the count of patent applications submitted by SMEs, encompassing various patent types such as inventions, designs, and utility models. This test aimed to scrutinize the impact of digital inclusive finance on the innovative endeavors of SMEs through an alternative metric. Secondly, in acknowledgment of the sway exerted by the COVID-19 pandemic, the scope of the dependent variable was confined to data until 2019, facilitating robustness testing within this constrained timeframe. The outcomes of these examinations are expounded in Table 6, and they exhibit a concurrence with the primary regression results. This congruence substantiates the coherence and dependability of the documented findings.

**Table 6:** Robustness test results.

VARIABLES	(1)	(2)
	INNOV	INNOV
DIFI	0.5354***	1.0480***
	(3.3051)	(4.8470)
size	0.5728***	0.1447**
	(12.1626)	(2.3937)
ROA	3.9674***	-5.9842***
	(6.0063)	(-6.0616)
GROW	-21.1464***	79.2486***
	(-2.9761)	(6.9793)
FAS	-92.7756***	-49.8273
	(-3.2581)	(-1.2620)
Age	-0.0002	-0.0197**
	(-0.0295)	(-2.3481)
Ind	0.0287	1.4188
	(0.0446)	(1.6341)
Dual	-0.0481	-0.0868
	(-0.7560)	(-1.0105)
Lev	0.8931***	-4.8683***
	(3.9445)	(-15.4019)
Constant	-11.5679***	-0.7278
	(-11.0824)	(-0.5592)
Observations	1,159	5,094
R-squared	0.463	0.432
Industry FE	YES	YES
Year FE	YES	YES

Robust t-statistics in parentheses

*** p<0.01

** p<0.05

* p<0.1

#### 4.5.2 Endogeneity treatment

To address potential concerns related to endogeneity and enhance the resilience of the findings, this study employs a set of strategies designed to mitigate bias stemming from endogeneity. Two principal sources of endogeneity are meticulously examined within the analysis. Firstly, the issue of reverse causality is addressed, wherein the technological innovation within SMEs might conceivably influence the progression of digital inclusive finance [65]. To counteract this potential bias, all explanatory variables at the enterprise level are aligned with a one-period temporal lag. This lagged approach serves to ameliorate the challenge of reverse causality by effectively accounting for the chronological sequence of events and consequently diminishing the likelihood of biased results. Secondly, the study duly acknowledges the conceivable presence of omitted variable bias, despite the incorporation of control variables such as year and industry during the model’s specification phase. Variables that elude measurement but retain the potential to exert influence can introduce distortion into the analysis. To rigorously evaluate the impact of digital inclusive finance on the technological innovation and advancement of SMEs, this paper employs the mobile phone penetration rate as an instrumental variable for the purpose of regression analysis. This instrumental variable methodology is employed to ascertain whether the outcomes of the original model are prone to bias arising from endogeneity concerns. By leveraging an instrumental variable that is hypothesized to exhibit a correlation with the endogenous variable (digital inclusive finance) while remaining unrelated to the error term, the study strives to derive unbiased estimates regarding the correlation between digital inclusive finance and technological innovation among SMEs.

Table 7 showcases the outcomes of the estimation process, revealing that the significant influence of digital inclusive finance on innovation within SMEs endures even subsequent to the implementation of measures to mitigate endogeneity bias. These measures encompass the application of a one-period lag to all explanatory variables and the utilization of instrumental variable regression. This observation substantiates that the impact of digital inclusive finance on SEM innovation remains pronounced, irrespective of the intricacies posed by endogeneity concerns. Hence, it accentuates the resilience of the inference that digital inclusive finance indeed plays a substantial and positive role in fostering innovation within the realm of SMEs.

**Table 7:** Endogeneity treatment results.

VARIABLES	(1)	(2)
	INNOVS	DIFI
L.DIFI	1.0414***	
	(4.9521)	
telephone		0.0041***
		(52.3729)
size	0.1651***	-0.0009
	(2.8036)	(-0.3199)
ROA	-6.5028***	0.0717*
	(-7.0477)	(1.7709)
GROW	83.4553***	-1.1900**
	(7.4270)	(-2.4300)
FAS	-37.6539	-17.6031***
	(-0.9400)	(-8.3739)
Age	-0.0111	-0.0003
	(-1.3484)	(-0.6919)
Ind	0.5923	0.1192***
	(0.7095)	(2.9019)
Dual	-0.0481	0.0232***
	(-0.5643)	(5.4423)
Lev	-5.0965***	0.0654***
	(-16.2116)	(4.5913)
Constant	-0.7937	-0.1018
	(-0.6231)	(-1.4455)
Observations	5,467	6,543
R-squared	0.442	0.952
Industry FE	YES	YES
Year FE	YES	YES

Robust t-statistics in parentheses

*** p<0.01

** p<0.05

* p<0.1

## 5 Discussion

Within this study, a fixed-effects model has been adeptly employed, with data spanning from 2011 to 2021, amalgamating the digital inclusive finance index with panel data encompassing SMEs listed on stock exchanges for empirical analysis. The resultant findings robustly substantiate the pivotal role played by digital inclusive finance in propelling SME innovation. Noteworthy facets such as coverage, depth of utilization, and the extent of digital service provision within the purview of digital inclusive finance all demonstrate a positive contribution to the advancement of innovation. This contribution is notably manifested through the alleviation of financing constraints encountered by SMEs, consequently fostering heightened levels of innovation activity. Heterogeneity analysis underscores that digital inclusive finance distinctly affects non-state-owned SMEs and high-tech SMEs with respect to their innovation endeavors. The preeminence of digital inclusive finance in elevating the innovation capacities of these specific categories of enterprises is a testament to its potential to redress conventional financial resource disparities. These segments, facing more acute financing constraints in comparison to others, observe a more substantial impetus towards innovation driven by digital inclusive finance. In light of these research outcomes, the following policy recommendations are posited:

**Firstly, cultivating healthy development and easing financing constraints:** At the government level, an active impetus should be lent to the cultivation of digital inclusive finance. This could entail encouraging the digitization and modernization of traditional financial services, accompanied by the provision of a diverse array of financial products and services meticulously tailored to the technological innovation and growth of SMEs. Financial institutions should embrace digital transformation, intensifying their support for digital technology to facilitate the expansion of inclusive finance. Convenient and reasonably priced financial services ought to be extended to SMEs, efficaciously mitigating their financing restrictions. Regulatory frameworks for digital inclusive finance should be refined, with a comprehensive comprehension of financial technology, collaborative regulatory mechanisms established, and prudent expansion of regulated digital inclusive finance ensured. This approach safeguards its status as a valuable instrument catering to the innovative financing aspirations of SMEs.

**Secondly, tailored policies for heterogeneous SME innovation: Discerning that** different enterprises grapple with varying financing challenges and that the influence of digital inclusive finance on innovation is divergent, targeted policies are recommended. A pronounced elevation in innovation levels is observed for non-state-owned and high-tech SMEs due to the pivotal role of digital inclusive finance in reconciling traditional financial resource discrepancies. Recognizing this, digital inclusive finance institutions should intimately acquaint themselves with the developmental idiosyncrasies of these enterprises and proffer bespoke financial products that ameliorate financing expenses, bolster innovation financing efficiency, and expedite innovation undertakings. For instance, harnessing digital technology for precise big data models, assessing the value of innovation projects, and enhancing SMEs’ access to financing can mitigate industry-based biases, thereby engendering a just financing milieu that stimulates innovation ardor.

Thirdly, augmenting SME self-improvement and prudent resource allocation: In an epoch characterized by rapid digital advancements, SMEs should enhance their adeptness in interfacing with digital inclusive finance to fuel their own progress. Augmenting enterprise operation and financial management systems, including comprehensive financial research and development project information disclosure, can alleviate financing impediments and streamline innovation financing efficiency. concurrently, SMEs should bolster their proficiency in leveraging digital inclusive finance. This necessitates bolstering financial literacy, making informed decisions about the allocation of digital inclusive finance resources, and cherry-picking fitting financial products and services in alignment with their distinct attributes and financing requisites.
