# Correction: The impact of digital inclusive finance on SMEs’ technological innovation activities—Empirical analysis based on the data of new third board enterprises

**DOI:** 10.1371/journal.pone.0296605

**Published:** 2024-01-02

**Authors:** Fuzhen Gu, Junguang Gao, Xiaoya Zhu, Jiamin Ye

There are errors in the Funding section. The correct Funding statement is: Sichuan Provincial Department of Science and Technology’s Soft Science Program project titled "Research on Fiscal Policies for the Effective Connection between Poverty Alleviation and Rural Revitalization in the Panxi Ethnic Region" (Project No. 2022JDR0078); F.G. Panzhihua University Doctoral Research Launch Funding Project, BKQJ2019075; The project from the Resource-Based City Development Research Center at Panzhihua University, a Key Research Base for Humanities and Social Sciences in Sichuan Province(ZYZX-ZD-2202).

The funders had no role in study design, data collection and analysis, decision to publish, or preparation of the manuscript.
